# When Standing Takes Your Breath Away: A Clinical Case of Platypnea-Orthodeoxia Syndrome

**DOI:** 10.7759/cureus.70677

**Published:** 2024-10-02

**Authors:** Mariana R Laranjeira, Rui P Dias, Catarina Rua, Catarina Dionísio, Gustavo Pires-Morais

**Affiliations:** 1 Internal Medicine, Vila Nova de Gaia/Espinho Hospital Center, Vila Nova de Gaia, PRT; 2 Family Medicine, Além D'Ouro, Vila Nova de Gaia, PRT; 3 Rheumatology, Vila Nova de Gaia/Espinho Hospital Center, Vila Nova de Gaia, PRT; 4 Interventional Cardiology, Vila Nova de Gaia/Espinho Hospital Center, Vila Nova de Gaia, PRT; 5 Cardiology, Hospital De Gaia, Gaia, PRT

**Keywords:** embolism, foramen ovale, paroxysmal dyspnea, platypnea-orthodeoxia syndrome, shunt

## Abstract

Platypnea-orthodeoxia syndrome (POS) is characterized by dyspnea due to a marked fall in blood oxygen saturation while assuming standing or sitting positions. It is a rare condition with an unknown prevalence. The triggering role may remain unclear in a considerable number of patients.

We present an 85-year-old female admitted to the emergency department (ED) due to aphasia, deviation of the right lip commissure, tachycardia, and dyspnea. History included a right femur shaft fracture in the previous month, and she was enrolled in a rehabilitation program. She already had a history of multiple falls and cerebrovascular disease. On physical examination, she was apyretic, normocardic, and normotensive, although tachypneic. Peripheral oxygen saturation was considerably different, being higher with the patient in the supine position when compared to a bed elevation of 30º. Lung fields were clear to auscultation, and cardiac examination showed no regurgitant heart murmurs or precordial impulses. No peripheral limb edema was noted. The neurologic examination was unremarkable. Her first arterial blood sample revealed hypoxemic respiratory failure. The remaining complementary diagnostic exams (CDE) were normal, apart from some old rib and spine fractures visible on a CT scan. The patient was admitted to the internal medicine ward for further investigation.

After a long march of CDE, a transesophageal echocardiogram revealed the cause of POS, a patent foramen ovale. A contrast test with agitated saline showed a significant passage of bubbles from the right atrium to the left during the Valsalva maneuver, confirming intracardiac right-left shunt, and also an aneurysmal dilatation and lipomatous hypertrophy of the interatrial septum. Percutaneous closure is the surgical treatment procedure used.

The intervention was discussed with the Department of Cardiology and a conservative approach was decided.

In recent years, increasing articles and case reports of POS have elicited the attention of physicians, who have acquired a greater awareness and have become more accurate in diagnosing and treating patients with unexplained or paroxysmal dyspnea. The challenge is to understand which are the higher-risk patients in order to refer them for primary percutaneous closure.

## Introduction

Platypnea-orthodeoxia syndrome (POS) is often a diagnostic challenge. It is characterized by dyspnea due to a marked fall in blood oxygen saturation while assuming standing or sitting positions. The causes include intracardiac shunts, pulmonary parenchymal ventilation/perfusion mismatch, and pulmonary arteriovenous shunts.

An anatomical heart defect may be responsible for most cases of POS, especially a patent foramen ovale (PFO), whether combined or not with structural or functional abnormalities of other thoracic or abdominal organs. Most people with PFO never develop symptoms because the pressure in the left atrium (LA) is slightly higher than in the right atrium (RA) and the atrial septum (AS) is functionally closed. The symptoms may develop when a flow phenomenon occurs, due to anatomical distortion, and/or because of an increased, although transient, pressure in the RA. This displacement is much more marked in the standing position, promoting an orthostatic preferential flow of desaturated blood through the area of septal discontinuity. 

Lung diseases also represent a significant cause of POS, especially those that entail a ventilation/perfusion mismatch -idiopathic pulmonary fibrosis and arteriovenous (AV) shunts at the lung bases. This is also frequent in patients with hepatopulmonary syndrome (HPS). Advanced liver disease is not required for HPS to develop, and the disease may worsen irrespective of hepatic function. It may also occur following a lung surgery or congenital and age-related thoracic malformations such as kyphoscoliosis [1-3].

In a considerable number of patients, the triggering factor for symptomatic disease may remain unclear despite an adequate diagnostic investigation.

## Case presentation

An 85-year-old female was admitted to the ED due to aphasia, deviation of the right lip commissure, tachycardia, and dyspnea, stated to be of at least 6 hours duration. There were no complaints of chest pain, cough, or other cardiorespiratory symptoms. Her previous medical history included a right femur shaft fracture one month prior, a previous transitory ischemic stroke 10 years ago, as well as dyslipidemia and multiple falls.

On physical examination, she had no fever, a regular pulse of 60 beats/min, a respiration rate of 22/min, and normal blood pressure. Without supplemental oxygen therapy, her saturation was 89% supine and 83% with a bed elevation of 30 degrees. Lung fields were clear to auscultation, and cardiac examination showed no regurgitant heart murmurs or palpable precordial impulses. She had no signs of pulmonary or peripheral congestion. The neurologic examination was unremarkable. Her first arterial blood sample with a fraction of inspired oxygen of 26% revealed hypoxemic respiratory failure. Blood tests, apart from a long-known normocytic normochromic anemia, were normal, and respiratory viruses, including SARS-COV-2, were negative. Her contrasted-chest computed tomography was unremarkable apart from some old rib and spine fractures (D7, D12, and L1). During her stay in the emergency department, she required supplemental oxygen, with a fraction of inspired oxygen of 31%, to maintain peripheral oxygen saturation above 94%. She was admitted to the internal medicine ward for further investigation.

**Figure 1:**
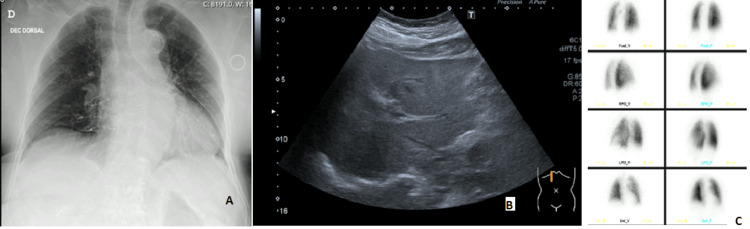
Patient's chest radiography (A), upper abdominal ultrasound (B), and CT pulmonary angiogram (C) The normality of the findings of these CDEs allowed the exclusion of post-pneumonectomy status, chronic liver disease, and pulmonary vascular diseases as causes of POS. CDE: complementary diagnostic exam; POS: platypnea-orthodeoxia syndrome

During her hospital stay, several causes of paroxysmal dyspnea were excluded [1-2]: 1. Post-pneumonectomy status and hydrothorax were immediately excluded, as the patient had no record of this surgical intervention and the chest X-ray revealed two well-expanded lungs of normal morphology (Figure 1A); 2. The absence of other respiratory symptoms, the normality of pulmonary auscultation, and the absence of a previous history of tobacco consumption or prolonged exposure to organic and inorganic particles allowed us to exclude pulmonary causes. The chest X-ray showed aortic artery calcification and mild bilateral reticular pattern with no clinical significance. N-terminal pro-B-type natriuretic peptide (NT-proBNP) was normal, excluding heart failure; 3. Hepatic function tests and upper abdominal ultrasound showed no evidence of cytocholestasis despite moderate hepatic steatosis. The patient had no ascites, jaundice, or encephalopathy so we excluded advanced chronic liver disease with HPS (Figure 1B); 4. Pulmonary embolism, arteriovenous malformations, or fistulae were excluded with a CT pulmonary angiogram and a lung ventilation-perfusion scan, respectively (Figure 1C).

Taking into account the patient's history of axial fragility fractures and her advanced age, significant kyphosis could explain this case. However, even though she had poor respiratory dynamics, there were no significant spinal deviations on clinical and imaging evaluation.

**Figure 2:**
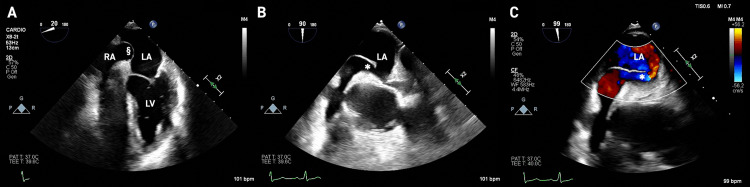
Patient's transesophageal echocardiogram The transesophageal echocardiogram revealed a patent foramen ovale. The contrast test with agitated saline confirmed the existence of an intracardiac right-left shunt. RA: right atrium, LA: left atrium, LV: left ventricle, *:* *foramen ovale

To assess the possibility of an intracardiac shunt, a transthoracic echocardiogram was done, showing dilatation of the ascending aorta and valvular degenerative changes without significant functional compromise [4]. Since transesophageal echocardiogram offers a superior visualization of posterior cardiac structures (particularly the LA), it was also performed, revealing a PFO (Figure 2). A contrast test with agitated saline also showed a significant passage of bubbles from the RA to the LA during the Valsalva maneuver, confirming the existence of an intracardiac right-left shunt. This finding is often accompanied by aneurysmal dilatation and lipomatous hypertrophy of the interatrial septum.

## Discussion

Percutaneous closure is the best treatment we can offer to our patients. Although we considered that the patient would benefit from this procedure, the Cardiology Department disagreed, taking into account the patient's frailty, motor limitations, and a late diagnosis of congenital heart disease without hemodynamic and/or functional impairment. 

For the reasons mentioned above, a conservative approach was chosen even though a conservative approach in elderly, frail patients also carries risks that should not be ignored. It was decided to prescribe long-term oxygen therapy as well as start anticoagulation, given the patient's high thromboembolic risk.

The decision between a minimally invasive procedure and a conservative approach requires the recognition of some clinical contexts that per se might confer an increased risk for significant disease and morbidity. For example, it has been found that patients with concurrent, spontaneous right-to-left shunt, anomalies of the interatrial septum, larger foramen ovale, and coagulation abnormalities - factor V mutation, protein C, S, and antithrombin deficiencies, anticardiolipin and antiphospholipid antibodies, and hyperhomocysteinemia - represent a subgroup at potentially higher risk for paradoxical embolism. Other temporary procoagulant states, such as oral contraceptives and prolonged immobilization, have also been correlated with a high stroke risk and paradoxical embolism. The size of any detected FO should be considered because of the higher risk of trans-septal passage of emboli for larger lesions [1,5]. Based on the presence/absence of these conditions, as well as symptoms of functional and hemodynamic impairment, the decision is made whether or not to proceed with closure. Possibly, in younger patients with fewer comorbidities, there would be no doubt regarding the treatment to choose. But as mentioned, it was a multidisciplinary decision in which we tried to offer the best to the patient and the clinical conditions she presented.

## Conclusions

In recent years, increasing articles and case reports on POS have elicited the attention of physicians, who have acquired a greater awareness and become more accurate in diagnosing and treating patients with unexplained or paroxysmal dyspnea. The challenge that remains is to understand which are the higher-risk patients in order to refer them for primary percutaneous closure, instead of a conservative approach.

Particularly, with this clinical case, we intend to alert to this clinical condition in order to optimize the proposed treatments as well as to expand the population in which they could be applied. We consider that being elderly should not be a limiting condition in the decision between a minimally invasive procedure with few associated risks and the institution of anticoagulation, which also carries risks, namely, a higher bleeding risk in these fragile patients.

## References

[1] Kerut E, Norfleet W, Plotnick G, Giles T (2020). Patent foramen ovale: a review of associated conditions and the impact of physiological size. J Am Coll Cardiol.

[2] Rigatelli G, Ronco F (2010). Platypnea-orthodeoxia syndrome: the diagnosis of unexplained hypoxia. J Am Coll Cardiol.

[3] De Vecchis R, Baldi C, Ariano C (2016). Platypnea-orthodeoxia syndrome: multiple pathophysiological interpretations of a clinical picture primarily consisting of orthostatic dyspnea. J Clin Med.

[4] Davison P, Clift PF, Steeds RP (2010). The role of echocardiography in diagnosis, monitoring closure and post-procedural assessment of patent foramen ovale. Eur J Echocardiogr.

[5] Rigatelli G, Dell'Avvocata F, Giordan M (2009). Embolic implications of combined risk factors in patients with patent foramen ovale (the CARPE criteria): consideration for primary prevention closure?. J Interv Cardiol.

